# Middle lobe syndrome associated with major haemoptysis

**DOI:** 10.1186/1749-8090-8-84

**Published:** 2013-04-15

**Authors:** Kasra Shaikhrezai, Maziar Khorsandi, Vipin Zamvar

**Affiliations:** 1 Department of cardiothoracic surgery, Royal infirmary of Edinburgh, 51 little France crescent, Old Dalkeith Rd, Edinburgh EH16 4SU, UK

**Keywords:** Middle lobe syndrome, Abnormal bronchial artery, Haemoptysis

## Abstract

A 60-year-old Indian woman who was suffering from recurrent pneumonia presented with major haemoptysis and a right-sided pleuritic chest pain. Initially the patient required resuscitation to optimise her haemodynamic parameters while oxygenation remained satisfactory. An urgent computed tomography pulmonary angiogram revealed right middle lobe syndrome which constitutes chronic collapse of the middle lobe accompanied by bronchiectatic changes. Angiography identified an abnormal bronchial artery and venous shunting which was embolised satisfactorily. Subsequently she underwent bronchoscopy which was unremarkable. Her post-operative course was uneventful and patient was discharged home. During the post-operative follow-up patient remained stable and was discharged from out-patient clinic after two years.

## Background

Middle lobe syndrome (MLS) is an uncommon disease involving the right middle lobe and/or left lingula. This syndrome which is characterised by the chronic collapse of the middle lobe and bronchiectasis [1] has been previously described in both adults and children [2,3]. The collapse is not usually associated with airway obstruction. Clinical manifestations are in consistence with that of pulmonary infection symptoms; that is cough as well as fever and less commonly dyspnoea, haemoptysis and pleuritic chest pain. Other reported histopathological features are chronic bronchitis with lymphoid hyperplasia, organising pneumonitis and granulomatous inflammation [1]. Although patients may remain asymptomatic, a wide spectrum of signs and symptoms has been reported [1].

## Case presentation

A 60-year-old Indian woman presented to the accident and emergency department with increasing haemoptysis. The onset of haemoptysis was acute with no associated cough. Although her oxygenation was satisfactory, she was tachycardic with heart rate of 109 beat-per-minute and blood pressure of 100/65 mmHg, categorising the haemorrhage as class 2 [4]. The bleeding was associated with a mild right sided pleuritic chest pain which was previously experienced particularly during the pneumonia episodes. Her past medical history included recurrent pneumonia for which she received various courses of antibiotics with no identified micro-organism. She also was screened for tuberculosis with negative results. The latest pneumonia pattern was radiologically in consistence with that of atypical pneumonia involving the middle zone of the right lung.

**Figure 1:**
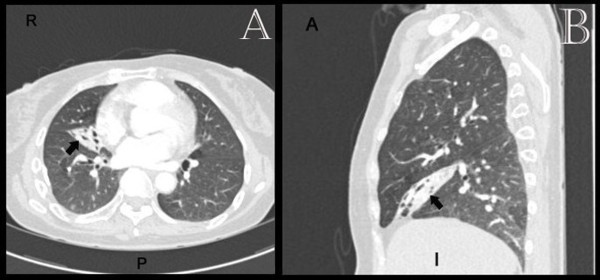
**CTPA (A:axial, B:sagittal): Right middle lobe collapse and bronchiectasis *(arrows)*.**

**Figure 2:**
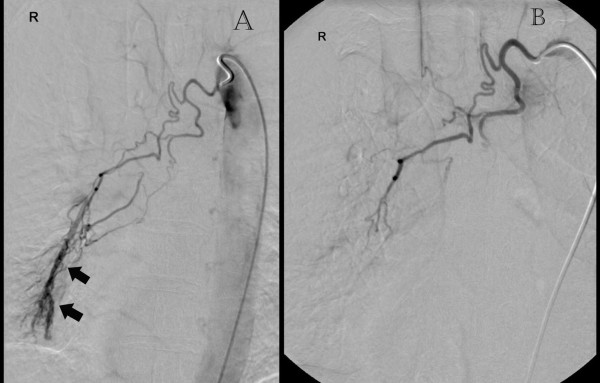
**(A) Angiography (pre-embolisation): Enlarged abnormal right bronchial artery with abnormal venous shunting (*arrows*).** (**B**) Post-embolisation.

Initially she was resuscitated with the fluids to stabilise the vital signs. The blood results including the coagulation profile and haemoglobin were unremarkable and within the normal limits. Immediately she underwent a computed tomography pulmonary angiogram (CTPA) which revealed right middle lobe collapse with bronchiectatic changes (Figure 1). Angiography identified an abnormal bronchial artery and venous shunting. The scheduled bronchoscopy was postponed proceeding with the radiological embolisation. The bronchial artery embolisation performed satisfactorily and the bleeding stopped completely (Figure 2). Thereafter she underwent bronchoscopy which was unremarkable with removal of some clots form the bronchial tree. Her post-operative course was satisfactory and patient was discharged home on her post-operative day two. Initially the patient was reviewed after 6 weeks, then one and two years in the out-patient clinic with the view to resecting the right middle lobe. As the patient remained stable with no complications throughout the follow-up and preferred not to undergo the surgery, at the end of follow-up she was discharged from the out-patient clinic.

## Conclusion

Haemoptysis is one of the manifestations of a patient with MLS [1]. It is estimated that 90% of haemoptysis originates from the bronchial tree circulation which may commonly be associated with anomalous bronchial artery [5]. Although in this case the malformation of the bronchial artery was ruled out, the chronic infection, bronchiectasis and atelectasis imposed an abnormal vasculature to the regional anatomy of the bronchial artery and its branches which can be labelled as a culprit for acute haemoptysis.

There is a consensus amongst clinicians that an urgent chest CT-scan should precede the bronchoscopy for haemoptysis investigation [5]. There is a paucity of evidence in the literature regarding the surgical management of MLS. A recent study on a limited number of children demonstrated that the surgical resection of the right middle lobe is an option when the medical therapy fails [6]. While more evidence is required for surgical resection of complicated or uncomplicated MLS, early diagnosis and embolisation remains the mainstay of haemoptysis management in these patients.

## Consent

Written informed consent was obtained from the patient for publication of this case report and accompanying images. A copy of the written consent is available for review by the Editor-in-Chief of this journal.

## Abbreviations

CT –scan: Computed tomography scan; CTPA: Computed tomography pulmonary angiogram; MLS: Middle lobe syndrome.

## Competing interests

The authors declare that they have no competing interests.

## Authors’ contributions

KS reviewed and resuscitated the patient and wrote the manuscript; MK conducted the investigations and literature search; VZ admitted the patient under his care, instructed and supervised the management. All authors read and approved the final manuscript.
